# Advanced lung cancer inflammation index and short-term mortality in sepsis: a retrospective analysis

**DOI:** 10.3389/fnut.2025.1563311

**Published:** 2025-05-14

**Authors:** Junjie Li, Yuekai Shao, Jie Zheng, Qiuyu Dai, Kun Yu, Song Qin, Xinxin Liu, Hong Mei

**Affiliations:** ^1^Department of Critical Care Medicine, Affiliated Hospital of Zunyi Medical University, Zunyi, China; ^2^Zunyi Medical University, Zunyi, China

**Keywords:** advanced lung cancer inflammation index (ALI), sepsis, 30-day all-cause mortality, 30-day ICU mortality, cox regression analysis

## Abstract

**Background:**

Sepsis is a notable cause of death and poor prognosis in the intensive care unit (ICU). Presents an ambiguous association between advanced lung cancer inflammation (ALI) and short-term lethality in sepsis patients. The purpose of this study is to explore this relationship.

**Methods:**

This retrospective study identified sepsis cases from the MIMIC-IV 3.0 dataset. Multivariable Cox regression analysis was used to evaluate the relationship between ALI and the risks of 30-day all-cause mortality (ACM) and ICU mortality. Kaplan–Meier (K-M) curves and log-rank tests were employed for survival analysis. Restricted cubic spline (RCS) regression was employed to explore the nonlinear association between ALI and mortality risk. Subgroup and sensitivity analyses were performed to confirm the reliability of the results and to evaluate the incremental effect of ALI on the prediction of short-term mortality.

**Results:**

A total of 4,147 sepsis cases were included in this study, with a 30-day ACM rate of 26.7% and a 30-day ICU mortality rate of 18.5%. In the completely adjusted Cox model, patients in the highest quartile of log2-ALI had a 38% lower risk of 30-day ACM (HR = 0.62, *p* < 0.001) and a 29% lower risk of 30-day ICU mortality (HR = 0.71, *p* = 0.002) compared to those in the lowest quartile. K-M curves showed that the group with the lowest log2-ALI had the lowest 30-day ACM and ICU survival rates (log-rank *p* < 0.001). RCS showed a nonlinear relationship between log2-ALI and 30-day ACM (P-overall < 0.001, P-nonlinear < 0.05). In all subgroups, the relationship between log2-ALI and outcomes showed no notable heterogeneity (P for interaction > 0.05), and four different sensitivity analyses yielded robust results. The combination of sequential organ failure assessment (SOFA) score and log2-ALI improved the predictive ability for 30-day ACM, with significant increases in C-statistic, Net Reclassification Improvement (NRI), and Integrated Discrimination Improvement (IDI).

**Conclusion:**

This research found that lower levels of ALI were notably linked to higher 30-day ACM and 30-day ICU mortality in sepsis patients, warranting further verification through prospective studies.

## 1 Introduction

Sepsis is a significant cause of mortality in ICU, with its high incidence, mortality rate, and substantial medical costs imposing a heavy burden on society (1). In 2017, it was estimated that there were 48.9 million cases of sepsis globally, with a mortality of 22.5%, representing nearly 20% of all global fatalities (2). Despite numerous advancements in treatment and critical care, the prognosis for sepsis remains poor due to its complexity and heterogeneity (3, 4). Early prognostic prediction and stratification of sepsis patients could facilitate timely and adequate interventions. Many sepsis biomarkers have been studied; however, their specificity and sensitivity are insufficient for clinical practice, limiting their application (5). Therefore, there is a need to explore more convenient and efficient sepsis biomarkers.

ALI is a novel composite index that combines inflammatory and nutritional status, including albumin, body mass index (BMI), and the neutrophil-to-lymphocyte ratio (NLR). Initially, ALI was used to evaluate the outcomes of tumor patients (6, 7), and later studies found it to be significantly associated with the outcomes of patients with hypertension, coronary artery disease, stroke, and heart failure (HF) (8–11). However, the relationship between ALI and sepsis prognosis remains unexplored.

This research aims to clarify the importance of ALI levels in the prognosis of sepsis patients.

## 2 Methods

### 2.1 Study population

The data utilized in this study were sourced from the MIMIC-IV dataset version 3.0, curated by the Massachusetts Institute of Technology (MIT). Medical data related to health status were extracted from the intensive care units at Beth Israel Deaconess Medical Center (BIDMC) located in Boston (12). During the data acquisition phase, Yuekai Shao (certificate number 59828695) was responsible for accessing and extracting the data. The Institutional Review Boards (IRBs) at BIDMC have approved the research protocol and have waived the need for explicit consent from the participating patients.

**Figure 1:**
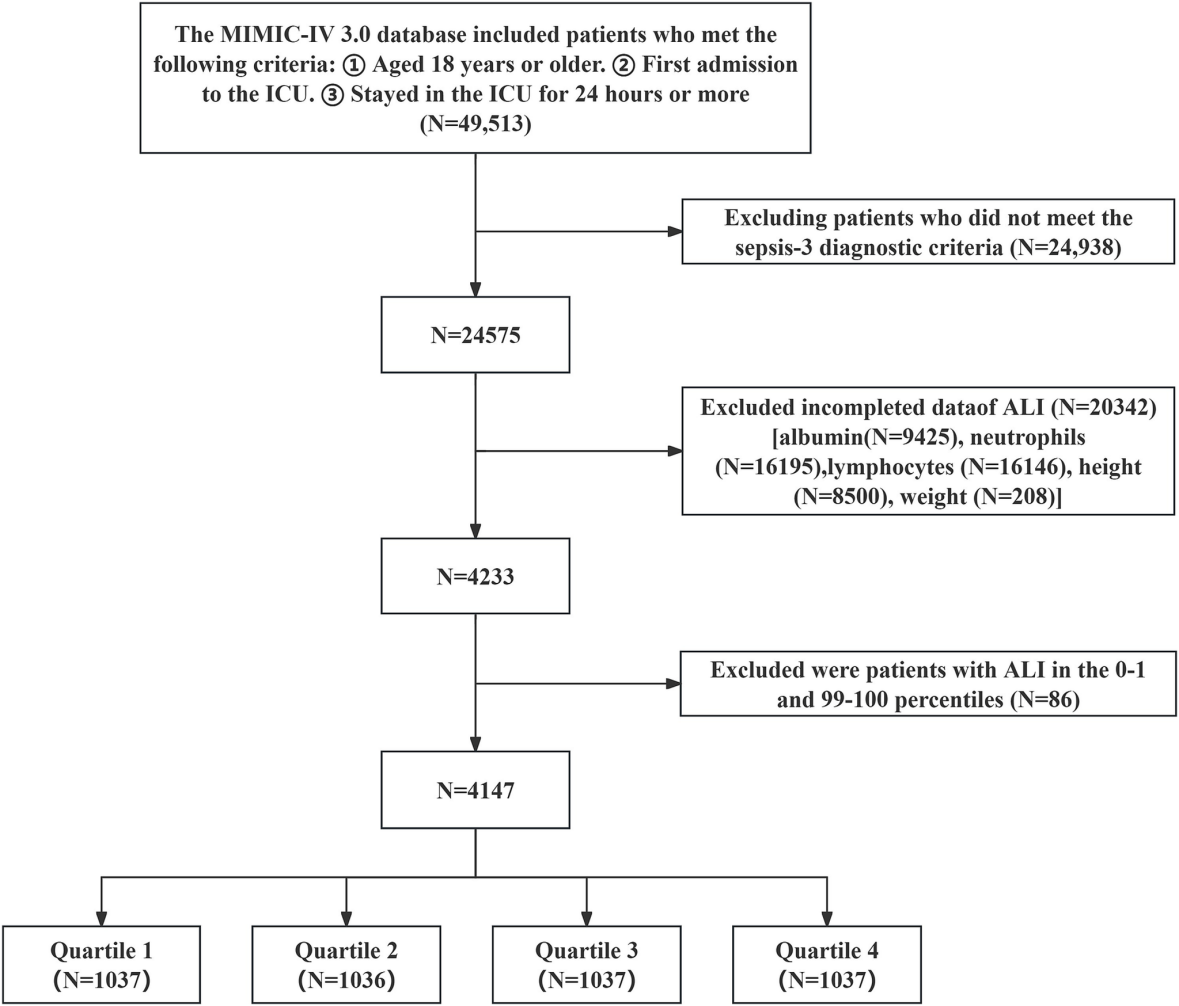
Patient selection flowchart.

The research included 49,513 individuals aged 18 years or older who had their first ICU admission and remained for at least 24 h. Patients not fulfilling the Sepsis-3 diagnostic criteria were excluded (3), as well as those with incomplete data on BMI, NLR, and serum albumin were excluded. Patients with ALI values in the 0th to 1st and 99th to 100th percentiles were also excluded. The final cohort consisted of 4,147 patients with sepsis, who were categorized into four different groups based on the quartiles of log2-ALI (Figure 1).

### 2.2 Acquisition of data

**Figure 2:**
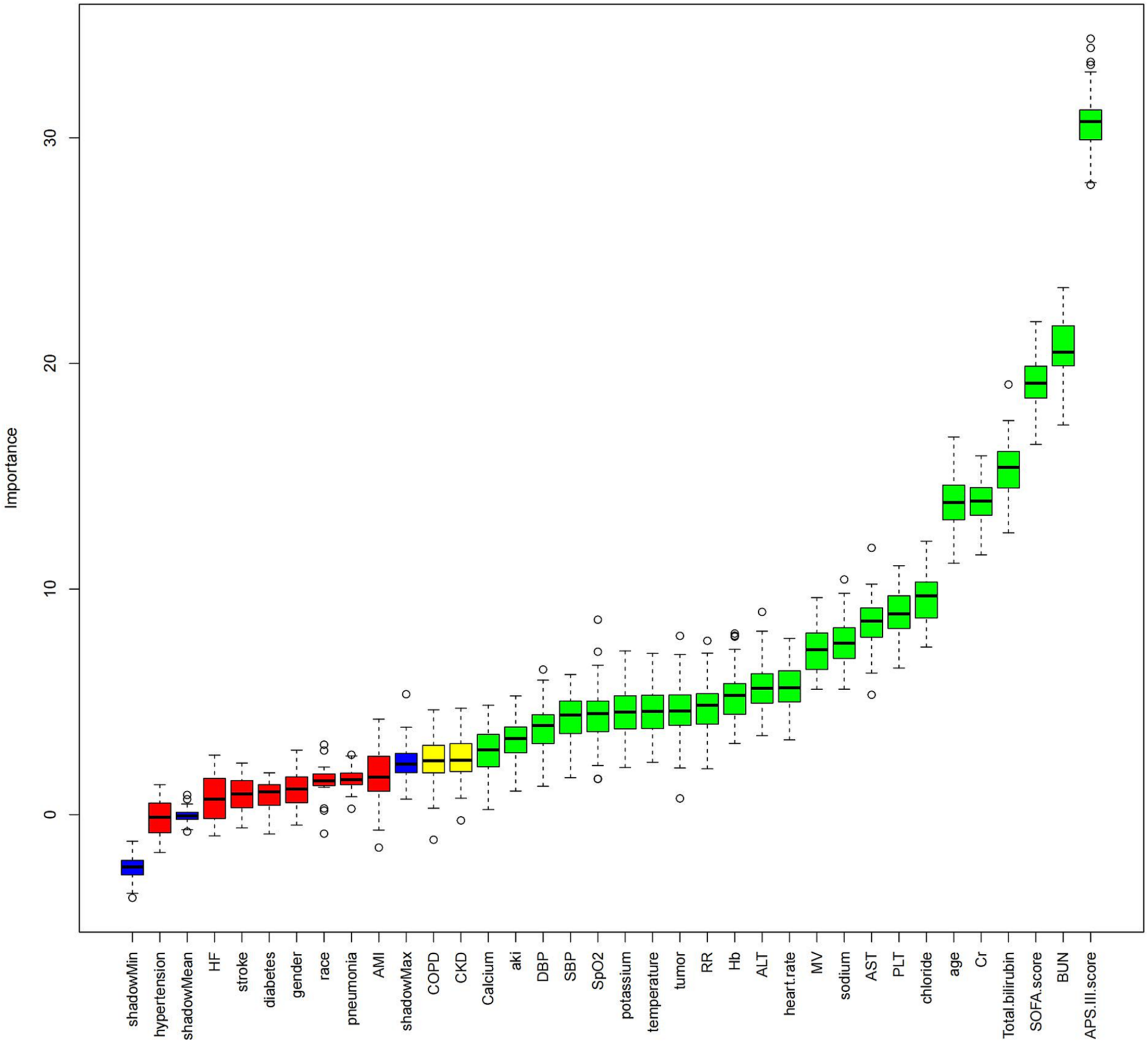
Boruta feature selection Image caption: The Boruta algorithm was used for feature selection to analyze the relationship between hemoglobin log2-ALI and 30-day ACM. The horizontal axis of the chart shows the names of the variables, while the vertical axis quantifies their importance. Box plots depict the importance of each variable in the model assessment, with green boxes representing key variables, yellow boxes representing potential variables, and red boxes indicating relatively unimportant variables. Variable classification was completed by comparing the importance scores of each variable with their corresponding shadow variables.

Data for sepsis cases were extracted from the MIMIC-IV 3.0 dataset using structured query language with Navicat Premium 16.0 software, including the following: (1) Demographic information: gender, age, race, BMI; (2) Initial vital signs recorded upon ICU admission: temperature, systolic blood pressure (SBP),heart rate, respiratory rate (RR), pulse oximetry oxygen saturation(SpO2), and diastolic blood pressure (DBP); (3) Initial laboratory tests recorded upon ICU admission: absolute counts of neutrophils and lymphocytes, hemoglobin (Hb), platelets (PLT), sodium, potassium, chloride, total bilirubin (TB), alanine aminotransferase (ALT), calcium, albumin (Alb), blood urea nitrogen (BUN), creatinine (Cr), aspartate aminotransferase (AST); (4) Comorbidities: diabetes, hypertension, stroke, chronic kidney disease (CKD), pneumonia, acute kidney injury (AKI), HF, chronic obstructive pulmonary disease (COPD), acute myocardial infarction (AMI), tumor; (5) Scores used in the study: Sequential Organ Failure Assessment (SOFA) score, Acute Physiology and Chronic Health Evaluation III (APS-III) score, and whether the patient received mechanical ventilation (MV) during ICU stay; (6) Outcomes: length of hospital stay, length of ICU stay, 30-day ACM, and 30-day ICU mortality. To maintain data reliability and minimize distortion caused by outliers, patients with ALI percentiles in the 0th to 1st and 99th to 100th ranges were excluded. Laboratory measurements with missing data exceeding 20% were not included in the analysis. Missing data were imputed using a random forest algorithm. Before investigating the relationship between log2-ALI values and ICU mortality, the Boruta machine learning algorithm was utilized for feature selection to assess the significance of each variable in the analytical model (13). This process involved assessing the distribution of feature importance scores and selecting predictors with significantly higher importance scores (Figure 2).

### 2.3 Exposure assessment

The ALI was computed using the formula: ALI = BMI (kg/m^2^) × albumin level (g/dL) / NLR (14). Subsequent statistics were log2 transformed to account for the right-skewed distribution of ALI (see Supplementary Figure S1).

### 2.4 Primary and secondary outcomes

The primary endpoint was 30-day ACM, with 30-day ICU mortality serving as the secondary endpoint.

### 2.5 Statistical analysis

Continuous variables are expressed as medians along with their interquartile ranges (IQR), while categorical variables are presented as numbers (percentages). Categorical variables were assessed using the Pearson chi-square test, whereas the Wilcoxon rank-sum test was utilized to compare continuous variables. To explore the relationship between log2-ALI and the risks of 30-day ACM and 30-day ICU mortality, we conducted multivariable Cox regression analysis to determine hazard ratios (HR) and assessed multicollinearity among variables using variance inflation factor (VIF) tests. Additionally, K-M curves were used for survival analysis, and log-rank tests were employed for comparisons. RCS was employed to explore potential nonlinear relationships between log2-ALI and mortality risk and to consider whether threshold analysis was necessary. In subgroup analyses, we conducted stratified analyses based on gender, age, race, BMI, and comorbidities. A series of sensitivity analyses were also performed, including (1) re-grouping log2-ALI by baseline tertiles; (2) excluding patients with tumors; (3) additionally adjusting white blood cell count (WBC) as a covariate; (4) using logistic regression for analysis. Finally, we evaluated the improvement in predictive ability for 30-day ACM by combining SOFA score with log2-ALI using C-statistic, NRI, and IDI.

All statistical analyses were conducted with R version 4.3.2 (R Foundation), with a two-tailed *p*-value threshold of less than 0.05 deemed to indicate statistical significance.

## 3 Results

### 3.1 Baseline characteristics

Table 1 shows the baseline characteristics of the 4,147 cases included in the final cohort, categorized by the quartiles of log2-ALI. There were 2,488 males (60.0%), with an average age of 65(54, 74) years. Compared to Q1, as log2-ALI increased, participants tended to be younger, with a lower prevalence of COPD, higher prevalence of hypertension, and lower levels of heart rate, neutrophils, NLR, ALT, BUN, Cr, SOFA score, and APS-III score. Conversely, weight, BMI, SBP, lymphocytes, SpO2, Alb, sodium, calcium, and chloride levels increased. Both 30-day ACM and 30-day ICU mortality decreased progressively.

**Table 1:** Patient baseline characteristics by ALI quartile grouping.

Variables	Overall, *N* = 4,147	Q1, *N* = 1,037	Q2, *N* = 1,036	Q3, *N* = 1,037	Q4, *N* = 1,037	*p*-value
Demographics						
Age, years	65 (54, 74)	67 (56, 75)	65 (53, 74)	64 (53, 73)	63 (52, 73)	<0.001
Male, *n* (%)	2,488 (60.0%)	632 (60.9%)	624 (60.2%)	633 (61.0%)	599 (57.8%)	0.385
White, *n* (%)	2,268 (54.7%)	581 (56.0%)	565 (54.5%)	574 (55.4%)	548 (52.8%)	0.500
Weight, Kg	82 (69, 98)	76 (64, 90)	80 (67, 94)	86 (72, 101)	89 (74, 107)	<0.001
Height, Cm	170 (163, 178)	170 (163, 178)	170 (163, 178)	170 (163, 178)	170 (163, 178)	0.353
BMI, Kg/m^2^	28 (24, 34)	26 (23, 31)	28 (24, 32)	29 (25, 35)	31 (26, 36)	<0.001
Vital signs						
Heart rate, bpm	91 (78, 107)	97 (81, 112)	92 (78, 107)	90 (78, 106)	87 (77, 103)	<0.001
DBP, mmHg	68 (58, 80)	67 (57, 79)	68 (58, 80)	69 (59, 81)	68 (57, 82)	0.110
SBP, mmHg	117 (102, 134)	113 (99, 132)	117 (102, 134)	118 (102, 135)	119 (104, 136)	<0.001
RR, bpm	20 (16, 25)	20 (17, 26)	20 (16, 25)	20 (16, 24)	20 (16, 24)	<0.001
Temperature, °C	36.89 (36.56, 37.22)	36.83 (36.50, 37.17)	36.89 (36.56, 37.22)	36.94 (36.56, 37.22)	36.89 (36.56, 37.22)	0.002
SpO_2,%_	98.0 (94.0, 100.0)	97.0 (94.0, 100.0)	97.0 (95.0, 100.0)	98.0 (95.0, 100.0)	98.0 (95.0, 100.0)	<0.001
Laboratory data						
Neutrophil, × 10^9^/L	10 (7, 15)	15 (11, 21)	11 (8, 16)	9 (7, 13)	7 (4, 9)	<0.001
Lymphocyte, × 10^9^/L	1.03 (0.61, 1.61)	0.52 (0.33, 0.81)	0.91 (0.63, 1.27)	1.24 (0.89, 1.71)	1.75 (1.20, 2.48)	<0.001
NLR	10 (5, 18)	28 (20, 43)	12 (10, 16)	7 (6, 9)	4 (3, 5)	<0.001
Hb, g/dL	10.40 (8.60, 12.30)	10.00 (8.40, 11.90)	10.40 (8.60, 12.13)	10.60 (8.80, 12.60)	10.60 (8.70, 12.40)	<0.001
PLT, ×10^9^ /L	182 (120, 252)	186 (119, 273)	182 (122, 250)	190 (126, 254)	167 (115, 235)	<0.001
Sodium, mmol/L	138.0 (135.0, 141.0)	138.0 (134.0, 141.0)	138.0 (135.0, 141.0)	138.0 (135.0, 142.0)	139.0 (136.0, 142.0)	<0.001
Potassium, mmol/L	4.20 (3.80, 4.70)	4.20 (3.80, 4.80)	4.30 (3.80, 4.70)	4.20 (3.80, 4.70)	4.20 (3.80, 4.70)	0.201
Chloride, mmol/L	103 (98, 107)	102 (97, 107)	103 (98, 107)	103 (99, 107)	104 (99, 108)	<0.001
Calcium, mg/dL	8.30 (7.70, 8.70)	8.10 (7.50, 8.60)	8.20 (7.70, 8.70)	8.30 (7.80, 8.80)	8.40 (7.90, 8.80)	<0.001
Total bilirubin, mg/dL	0.70 (0.40, 1.50)	0.80 (0.40, 1.90)	0.70 (0.40, 1.70)	0.70 (0.40, 1.30)	0.60 (0.40, 1.20)	<0.001
ALT, IU/L	33 (18, 79)	37 (19, 88)	35 (18, 91)	33 (18, 77)	29 (16, 65)	<0.001
Albumin, g/dL	2.90 (2.50, 3.30)	2.60 (2.30, 3.00)	2.80 (2.40, 3.30)	3.00 (2.70, 3.40)	3.20 (2.90, 3.60)	<0.001
AST, IU/L	50 (27, 127)	52 (28, 133)	52 (28, 153)	49 (27, 124)	46 (26, 103)	0.002
BUN, mg/dL	23 (14, 39)	28 (17, 49)	25 (15, 43)	20 (14, 34)	19 (13, 31)	<0.001
Cr, mg/dL	1.20 (0.80, 1.90)	1.40 (0.80, 2.30)	1.20 (0.80, 2.10)	1.10 (0.80, 1.70)	1.00 (0.80, 1.60)	<0.001
ALI, U	9 (4, 17)	3 (2, 3)	6 (5, 8)	12 (10, 14)	26 (20, 36)	<0.001
log2-ALI, U	3.15 (2.13, 4.05)	1.36 (0.80, 1.79)	2.66 (2.38, 2.92)	3.59 (3.36, 3.82)	4.68 (4.34, 5.18)	<0.001
Complication, *n* (%)						
Hypertension	1,417 (34.2%)	321 (31.0%)	324 (31.3%)	365 (35.2%)	407 (39.2%)	<0.001
Diabetes	1,292 (31.2%)	273 (26.3%)	315 (30.4%)	353 (34.0%)	351 (33.8%)	<0.001
AKI	3,849 (92.8%)	942 (90.8%)	968 (93.4%)	974 (93.9%)	965 (93.1%)	0.034
HF	1,295 (31.2%)	340 (32.8%)	316 (30.5%)	336 (32.4%)	303 (29.2%)	0.257
CKD	887 (21.4%)	231 (22.3%)	251 (24.2%)	212 (20.4%)	193 (18.6%)	0.013
Stroke	241 (5.8%)	58 (5.6%)	59 (5.7%)	55 (5.3%)	69 (6.7%)	0.583
Pneumonia	1,911 (46.1%)	497 (47.9%)	501 (48.4%)	471 (45.4%)	442 (42.6%)	0.033
COPD	599 (14.4%)	183 (17.6%)	139 (13.4%)	139 (13.4%)	138 (13.3%)	0.009
AMI	735 (17.7%)	191 (18.4%)	183 (17.7%)	189 (18.2%)	172 (16.6%)	0.695
Tumor	499 (12.0%)	177 (17.1%)	105 (10.1%)	117 (11.3%)	100 (9.6%)	<0.001
Others						
MV	3,890 (93.8%)	961 (92.7%)	979 (94.5%)	978 (94.3%)	972 (93.7%)	0.306
SOFA, score	7.0 (4.0, 10.0)	8.0 (5.0, 11.0)	8.0 (5.0, 11.0)	7.0 (4.0, 9.0)	7.0 (4.0, 9.0)	<0.001
APS-III, score	52 (39, 69)	59 (46, 77)	55 (42, 74)	49 (37, 64)	45 (33, 61)	<0.001
Outcomes						
Length of hospital stay	17 (9, 29)	17 (9, 28)	18 (10, 30)	18 (10, 30)	16 (9, 28)	0.006
Length of ICU stay	7 (4, 14)	7 (4, 13)	8 (4, 15)	7 (4, 14)	7 (3, 14)	0.005
30-day all-cause mortality	1,107 (26.7%)	400 (38.6%)	300 (29.0%)	211 (20.3%)	196 (18.9%)	<0.001
30-day ICU mortality	768 (18.5%)	268 (25.8%)	208 (20.1%)	151 (14.6%)	141 (13.6%)	<0.001

Data are shown as the median (interquartile range, IQR) or as numbers (percentages). SBP systolic blood pressure, SpO2 pulse oximetry oxygen saturation, RR respiratory rate, DBP diastolic blood pressure, TB total bilirubin, ALT alanine aminotransferase, Hb hemoglobin, Alb albumin, BUN blood urea nitrogen, Cr creatinine, PLT platelets, AST aspartate aminotransferase, CKD chronic kidney disease, COPD chronic obstructive pulmonary disease, AKI acute kidney injury, HF heart failure, AMI acute myocardial infarction, SOFA Sequential Organ Failure Assessment score, APS-III Acute Physiology and Chronic Health Evaluation III score, MV mechanical ventilation.

### 3.2 Cox regression analysis of log2-ALI and mortality

Table 2 shows that multivariable Cox regression models revealed a notable association between log2-ALI and both 30-day ACM and 30-day ICU mortality in sepsis cases. In the completely adjusted model (Model 3), when log2-ALI was treated as a continuous variable, each one-unit increase in log2-ALI was linked to a 13% decrease in 30-day ACM (HR = 0.87, *p* < 0.001) and a 9% decrease in 30-day ICU mortality (HR = 0.91, *p* < 0.001). Upon categorizing log2-ALI into quartiles, compared to Q1, patients in Q2 to Q4 had notably lower 30-day ACM and 30-day ICU mortality (P for trend < 0.001). In particular, patients in the highest quartile had a 38% lower 30-day ACM (HR = 0.62, *p* < 0.001) and a 29% lower 30-day ICU mortality (HR = 0.71, *p* = 0.002).

**Table 2:** Multivariate cox regression analysis.

Variables	Model 1		Model 2		Model 3	
	HR(95% CI)	*p*-value	HR(95% CI)	*p*-value	HR(95% CI)	*p*-value
30-day all-cause mortality						
log2-ALI (continuous)	0.79(0.76, 0.82)	<0.001	0.80(0.76, 0.83)	<0.001	0.87(0.83, 0.91)	<0.001
log2-ALI (quartiles)						
Quartile 1	Ref		Ref		Ref	
Quartile 2	0.71(0.61, 0.82)	<0.001	0.72(0.62, 0.84)	<0.001	0.80(0.69, 0.93)	0.004
Quartile 3	0.47(0.40, 0.56)	<0.001	0.48(0.41, 0.57)	<0.001	0.63(0.53, 0.75)	<0.001
Quartile 4	0.44(0.37, 0.52)	<0.001	0.45(0.38, 0.53)	<0.001	0.62(0.52, 0.75)	<0.001
*P* for trend		<0.001		<0.001		<0.001
30-day ICU mortality						
log2-ALI (continuous)	0.82(0.78, 0.86)	<0.001	0.82(0.78, 0.87)	<0.001	0.91(0.87, 0.96)	<0.001
log2-ALI (quartiles)						
Quartile 1	Ref		Ref		Ref	
Quartile 2	0.75(0.63, 0.90)	0.002	0.76(0.64, 0.91)	0.003	0.84(0.70, 1.01)	0.070
Quartile 3	0.53(0.43, 0.64)	<0.001	0.54(0.44, 0.66)	<0.001	0.73(0.60, 0.90)	0.003
Quartile 4	0.49(0.40, 0.60)	<0.001	0.50(0.41, 0.61)	<0.001	0.71(0.58, 0.89)	0.002
*P* for trend		<0.001		<0.001		<0.001

Model 1: Unadjusted. Model 2: Adjusted by age, gender, and race. Model 3: Adjusted by age, gender, race, heart rate, SBP, DBP, RR, SpO2, temperature, Hb, PLT, sodium, potassium, calcium, chloride, Total bilirubin, ALT, AST, BUN, Cr, aki, COPD, tumor, CKD, MV, SOFA score, APS-III score.

### 3.3 K-M survival curves

**Figure 3:**
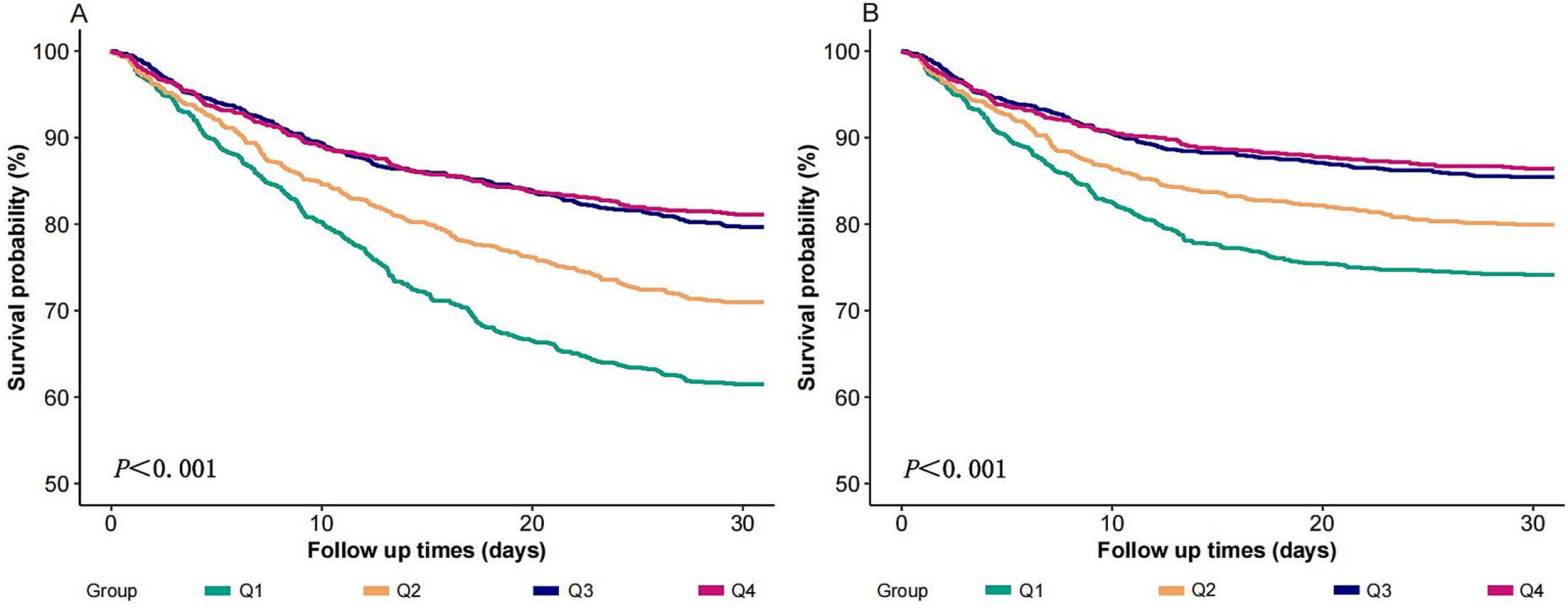
K-M chart of mortality by the log2-ALI. **(A)** 30-day ACM; **(B)** 30-day ICU mortality.

Figure 3 shows that cases with the Q1 (lowest log₂-ALI quartile) had the lowest 30-day all-cause survival rates and 30-day ICU survival rates compared to those with higher log2-ALI levels (log-rank *p* < 0.001).

### 3.4 Results of fitting curves

**Figure 4:**
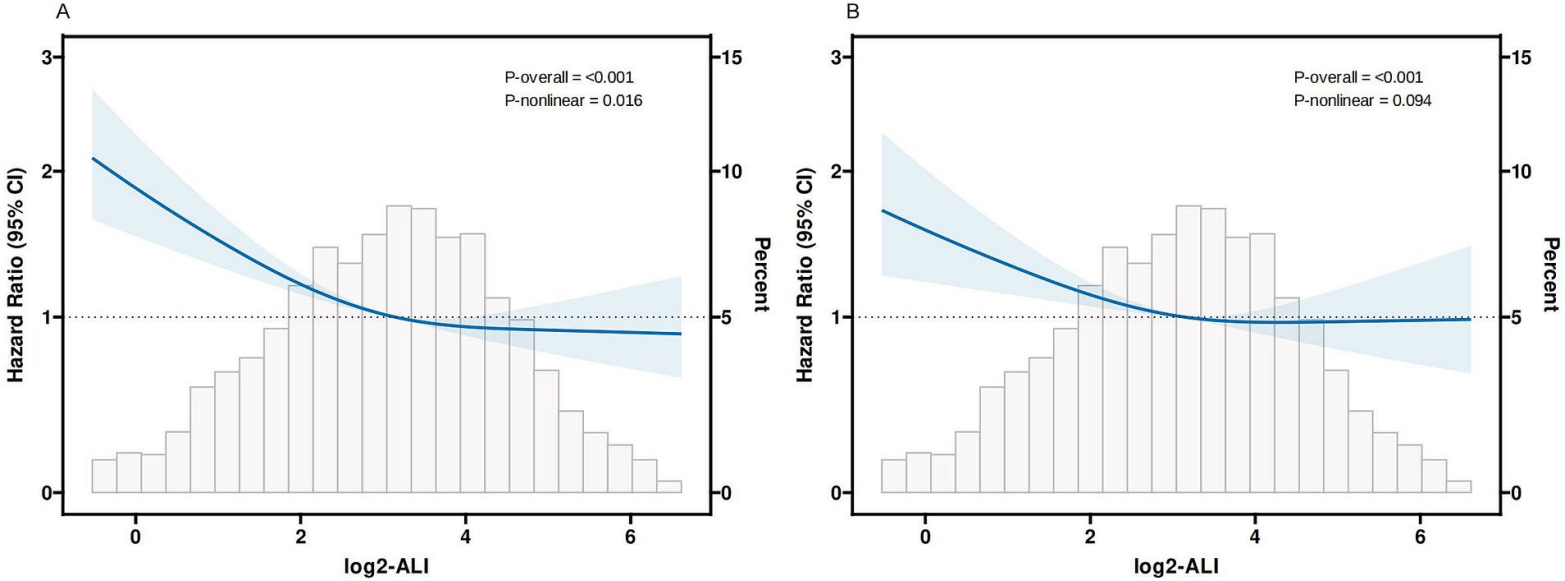
Curves fitting log2-ALI to illustrate its correlation with mortality outcomes **(A)** 30-day ACM; **(B)** 30-day ICU mortality Adjusted by age, gender, race, heart rate, SBP, DBP, RR, SpO2, temperature, Hb, PLT, sodium, potassium, calcium, chloride, Total bilirubin, ALT, AST, BUN, Cr, aki, COPD, tumor, CKD, MV, SOFA score, APS-III score.

In the completely adjusted model, RCS demonstrated a nonlinear relationship between log2-ALI and 30-day ACM (P-overall < 0.001, P-nonlinear < 0.05), whereas no significant nonlinear relationship was observed for ICU 30-day mortality (P-overall < 0.001, P-nonlinear > 0.05) (Figure 4). Additional analysis of threshold effects revealed that log2-ALI exhibits a saturation effect at an inflexion point of 4.265. The inverse association with 30-day ACM was more pronounced prior to log2-ALI reaching 4.265. Beyond this inflexion point, the impact on reducing the risk of 30-day ACM became insignificant. However, the difference did not reach statistical significance (Log likelihood ratio, *p* > 0.05) (see Supplementary Table S1).

### 3.5 Subgroup analysis

**Figure 5:**
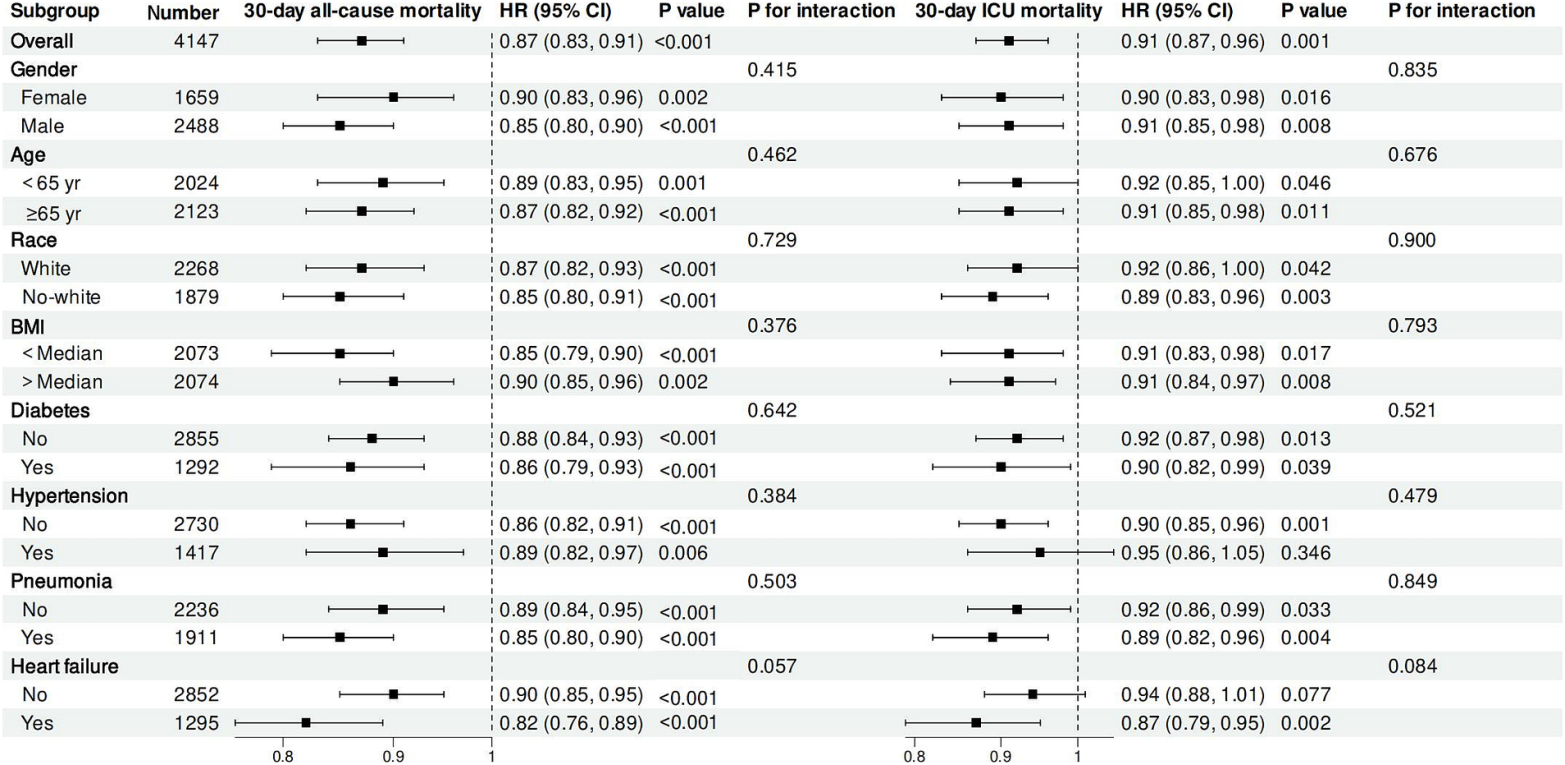
Subgroup analysis.

We investigated the possible variations in the relationship between log2-ALI and 30-day ACM and 30-day ICU mortality among sepsis cases across different subgroups. The findings indicated no notable differences in these relationships across age, gender, race, BMI, hypertension, diabetes, pneumonia, and HF (P for interaction > 0.05) (Figure 5).

### 3.6 Sensitivity analysis

Cases were initially divided into three categories according to the tertiles of log2-ALI and re-analyzed using Cox regression. The findings revealed that after comprehensive adjustment when log2-ALI was categorized, patients in the highest tertile exhibited a 36% reduced 30-day ACM (HR = 0.64, *p* < 0.001) and a 29% reduced 30-day ICU mortality (HR = 0.71, *p* < 0.001) compared to the first tertile (see Supplementary Table S2).

Subsequently, after excluding patients with tumors, the cohort was reduced to 3,648 participants. When log2-ALI was categorized in Model 3, patients in the highest quartile showed a 41% lower 30-day ACM (HR = 0.59, *p* < 0.001) and a 34% lower 30-day ICU mortality (HR = 0.66, p < 0.001) compared to the lowest quartile (see Supplementary Table S3).

Furthermore, adjustments were made for white blood cell count (WBC). When categorized, in Model 3, patients in the highest quartile had a 38% lower 30-day ACM (HR = 0.62, *p* < 0.001) and a 29% lower 30-day ICU mortality (HR = 0.71, *p* = 0.002) compared Q1 (see Supplementary Table S4).

Ultimately, logistic regression analysis was employed. The results showed that with all variables adjusted, with log2-ALI as a continuous variable, each one-unit increase in log2-ALI was linked to a 19% decrease in 30-day ACM (Odds Ratio [OR] = 0.81, *p* < 0.001) and a 13% decrease in 30-day ICU mortality (OR = 0.87, *p* < 0.001). When categorized, patients in the highest quartile had a 51% lower 30-day ACM (OR = 0.49, *p* < 0.001) and a 39% lower 30-day ICU mortality (OR = 0.61, *p* < 0.001) compared to Q1 (see Supplementary Table S5). Overall, the results of the sensitivity analyses were robust.

### 3.7 Incremental value of log2-ALI to SOFA score for predicting 30-day ACM

The SOFA score is a significant tool for evaluating the severity of illness and forecasting mortality in sepsis patients. As shown in Table 3, we calculated the C-statistic, NRI, and IDI to evaluate the incremental predictive value of log2-ALI for 30-day ACM. The addition of log2-ALI enhanced the prognostic value of the SOFA score for 30-day ACM. The C-statistic significantly increased from 0.656 to 0.686 (*p* < 0.001). After adding log2-ALI, both NRI and IDI significantly increased [continuous NRI (95% CI): 0.014 (0.007, 0.023), *p* < 0.001; IDI (95% CI): 0.149 (0.116, 0.183), p < 0.001] (Table 3).

**Table 3:** Incremental prognostic value of log2-ALI for 30-day ACM.

Statistical measure	SOFA score	SOFA score+log2-ALI	*p*-value
C-statistic (95% CI)	0.656 (0.637, 0.676)	0.686 (0.667, 0.704)	<0.001
Continuous NRI (95% CI)	Ref	0.014 (0.007, 0.023)	<0.001
IDI (95% CI)	Ref	0.149 (0.116, 0.183)	<0.001

## 4 Discussion

This research offers fresh perspectives on the significance of ALI in predicting the outcomes of sepsis patients. Our results reveal a notable association between lower levels of ALI and higher 30-day ACM and 30-day ICU mortality, indicating that the study suggests that ALI could function as a potential prognostic indicator for early risk stratification and prognostic evaluation. This association remained robust across various subgroup analyses and sensitivity analyses, showing that our findings are consistent and reliable. The combination of SOFA score and log2-ALI further enhanced the ability to predict the risk of death in sepsis cases. Given the high mortality associated with sepsis and the need for more effective prognostic tools, these results are particularly relevant for guiding clinical decision-making and resource allocation.

Sepsis is a critical, life-endangering syndrome marked by organ failure, which is closely related to inflammation in its aetiology, progression, and prognosis (15). In the initial acute response of the body to invasive pathogens, macrophages phagocytose pathogens and produce pro-inflammatory cytokines, triggering a cytokine storm and activating the innate immune system (16, 17). Inflammation and its advancement are often accompanied by a decrease in lymphocyte count and an increase in neutrophil count, although the reduction in lymphocyte count may be delayed during inflammation (18). Moreover, in cachexia and other certain diseases, the neutrophil count may not increase during inflammation (19). Therefore, the NLR, which takes both into account, is a more reliable prognostic indicator of acute inflammation. A meta-analysis by Wu et al. indicated that NLR can be used to forecast the prognosis and mortality risk in adult sepsis cases (20).

Malnutrition poses a risk for unfavorable outcomes in sepsis (21). The acute catabolic response in sepsis leads to the rapid activation of the body’s energy stores, with concurrent breakdown of stores of muscle, glycogen, and lipids to promote glucose production, which can result in muscle atrophy, weight loss, and loss of physical function (22, 23). Serum albumin, as a key marker of nutritional status, is inversely related to the severity of sepsis in patients (24, 25). Sepsis patients with hypoalbuminemia have a higher risk of experiencing renal failure, prolonged ICU stay, extended MV, and increased hospital mortality (26). Notably, inflammatory mediators in sepsis serve as potent catalysts for catabolism; for instance, cytokines are crucial in muscle protein degradation, stimulate bone resorption, and induce lipolysis in adipocytes (27, 28).

Although the role of obesity in the outcome of sepsis cases remains controversial, a meta-analysis targeting BMI has shown that being overweight is associated with lower mortality in sepsis (29). The “obesity paradox” admits to multiple explanatory avenues. Initially, increased body mass furnishes a reservoir of nutrients, with surplus adiposity acting as a vital energy depot during critical illness (30, 31). Moreover, adipose tissue modulates immune responses through the release of substances like leptin, a cytokine with anti-inflammatory properties (32). Bornstein et al. observed that among patients with acute sepsis, those who survived exhibited average plasma leptin concentrations threefold higher than those who did not survive (33).

ALI is an indicator used to comprehensively assess a patient’s nutritional and inflammatory status. Given that inflammation frequently results in hypoalbuminemia and reduced BMI, prior research has integrated these indicators with inflammatory markers to forecast the outcomes of cases with tumors (6, 7). Subsequently, ALI has also been effectively used in predicting the prognosis of cases of hypertension, HF, coronary artery disease, and stroke (8–11). As of now, no studies have examined the correlation between ALI and mortality among sepsis. In our study, we first found that an increase in ALI was significantly associated with a decrease in short-term mortality in sepsis. This suggests to clinicians that maintaining ALI within an appropriate range for sepsis patients (for example, through managing weight, supplementing albumin, and administering anti-inflammatory therapies) is crucial for prognosis. Monitoring the fluctuations in ALI can also aid in setting individualized ALI benchmarks, thereby optimizing the short-term survival prospects for sepsis patients.

Although the results of our study are robust, it is important to recognize several limitations. Firstly, the study’s retrospective nature constrains our capacity to ascertain a causal connection between ALI and the outcomes of sepsis. Second, the data were derived from the MIMIC-IV dataset, which may not fully represent sepsis patients in other regions or settings, potentially affecting the generalizability of our results. Third, this study relied on a single measurement of ALI at baseline, which may not capture the dynamic changes in patients’ inflammatory and nutritional status over time. Fourth, despite adjusting for numerous potential confounders, the possibility of residual confounding remains due to factors that were unmeasured or unknown. Finally, this study did not include an external validation cohort, which is necessary to confirm the stability and applicability of our findings in different populations. Future prospective studies are needed to validate ALI’s prognostic value in sepsis.

## 5 Conclusion

Our study indicates that lower levels of ALI were notably associated with higher 30-day ACM and 30-day ICU mortality in patients with sepsis, suggesting ALI’s potential as a prognostic indicator. Further research is needed to validate these findings and elucidate the underlying mechanisms.

## Data Availability

Publicly available datasets were analyzed in this study. This data can be found here: https://www.physionet.org/content/mimiciv/3.0/.
