# Supplementary material for: Advanced lung cancer inflammation index and short-term mortality in sepsis: a retrospective analysis
Source: Front Nutr. 2025 May 14;12:1563311. doi: 10.3389/fnut.2025.1563311 (PMC12116343; doi:10.3389/fnut.2025.1563311)
Supplement: Supplementary file 1 [file Data_Sheet_1.DOCX]

Supplementary Material

# Supplementary Figures

## Supplementary S1


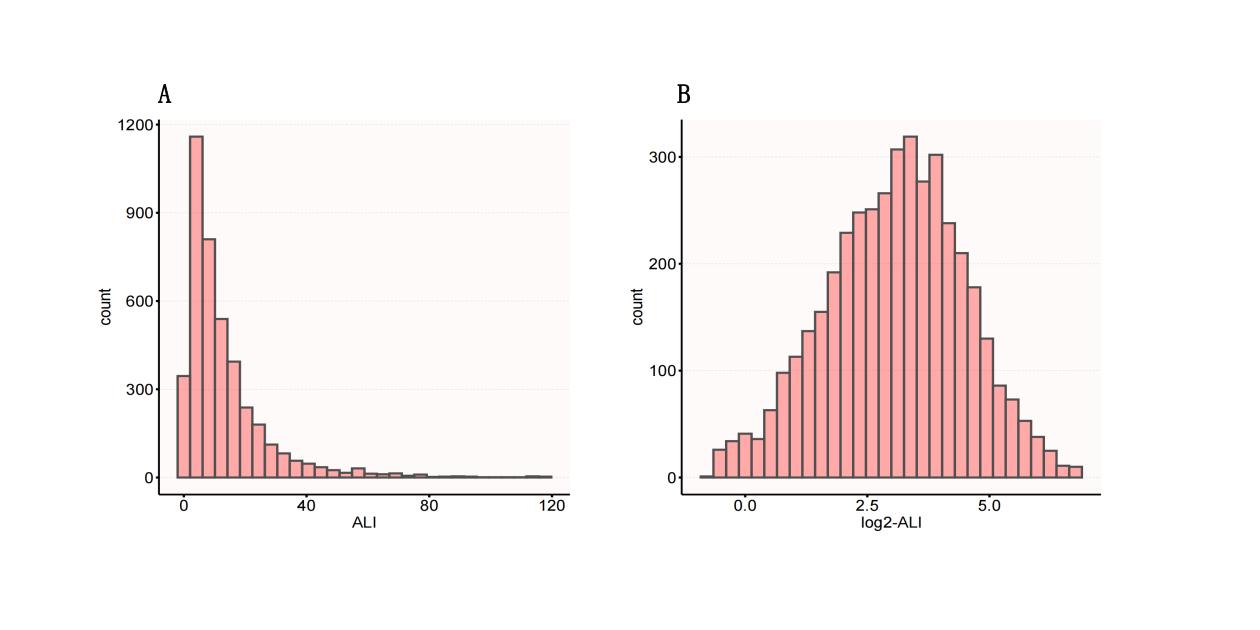


Fig. S1 ALI was transformed using log2

# Supplementary Tables

## Supplementary Table S1

**Table S1** Threshold Analysis

| sepsis | Adjust HR(95% CI) | P-value |
| --- | --- | --- |
| Fitting by the standard COX regression model | 0.87（0.83, 0.91） | <0.001 |
| Fitting by the two-piecewise COX regression model |  |  |
| log2-ALI |  |  |
| inflection point | 4.265 |  |
| log2-ALI＜4.265 | 0.83 (0.78,0.87) | <0.001 |
| log2-ALI＞4.265 | 1.09 (0.78,1.52) | 0.607 |
| Log likelihood ratio |  | 0.355 |

Adjusted by age, gender, race, heart rate, SBP, DBP, RR, SpO2, temperature, Hb, PLT, sodium, potassium, calcium, chloride, Total bilirubin, ALT, AST, BUN, Cr, aki, COPD, tumor, CKD, MV, SOFA score, APS-III score

## Supplementary Table S2

**Table S2** Sensitivity Analysis -1

| **Variables** | **Model 1** | | **Model 2** | | **Model 3** | |
| --- | --- | --- | --- | --- | --- | --- |
|  | **HR(95% CI)** | **P-value** | **HR(95% CI)** | **P-value** | **HR(95% CI)** | **P-value** |
| **30-day all-cause mortality** |  |  |  |  |  |  |
| log2-ALI (continuous) | 0.79（0.76, 0.82） | <0.001 | 0.80（0.76, 0.83） | <0.001 | 0.87（0.83, 0.91） | <0.001 |
| log2-ALI (tertiles) |  |  |  |  |  |  |
| Tertile 1 | Ref |  | Ref |  | Ref |  |
| Tertile 2 | 0.65(0.57, 0.75) | <0.001 | 0.66(0.58, 0.76) | <0.001 | 0.77(0.67, 0.88) | <0.001 |
| Tertile 3 | 0.46(0.39, 0.53) | <0.001 | 0.47(0.40, 0.55) | <0.001 | 0.64(0.54, 0.75) | <0.001 |
| P for trend |  | <0.001 |  | <0.001 |  | <0.001 |
| **30-day ICU mortality** |  |  |  |  |  |  |
| log2-ALI (continuous) | 0.82（0.78, 0.86） | <0.001 | 0.82（0.78, 0.87） | <0.001 | 0.91（0.87, 0.96） | <0.001 |
| log2-ALI (tertiles) |  |  |  |  |  |  |
| Tertile 1 | Ref |  | Ref |  | Ref |  |
| Tertile 2 | 0.68(0.58, 0.80) | <0.001 | 0.69(0.59, 0.82) | <0.001 | 0.81(0.69, 0.96) | 0.017 |
| Tertile 3 | 0.50(0.42, 0.60) | <0.001 | 0.50(0.42, 0.60) | <0.001 | 0.71(0.58, 0.85) | <0.001 |
| P for trend |  | <0.001 |  | <0.001 |  | <0.001 |

Model 1: Unadjusted.

Model 2: Adjusted by age, gender, and race

Model 3: Adjusted by age, gender, race, heart rate, SBP, DBP, RR, SpO2, temperature, Hb, PLT, sodium, potassium, calcium, chloride, Total bilirubin, ALT, AST, BUN, Cr, aki, COPD, tumor, CKD, MV, SOFA score, APS-III score

## Supplementary Table S3

**Table S3** Sensitivity Analysis -2

| **Variables** | **Model 1** | | **Model 2** | | **Model 3** | |
| --- | --- | --- | --- | --- | --- | --- |
|  | **HR(95% CI)** | **P-value** | **HR(95% CI)** | **P-value** | **HR(95% CI)** | **P-value** |
| **30-day all-cause mortality** |  |  |  |  |  |  |
| log2-ALI (continuous) | 0.78（0.74, 0.82） | <0.001 | 0.79（0.75, 0.82） | <0.001 | 0.86（0.82, 0.90） | <0.001 |
| log2-ALI (quartiles) |  |  |  |  |  |  |
| Quartile 1 | Ref |  | Ref |  | Ref |  |
| Quartile 2 | 0.69(0.59, 0.81) | <0.001 | 0.71(0.60, 0.83) | <0.001 | 0.79(0.67, 0.93) | 0.005 |
| Quartile 3 | 0.46(0.38, 0.55) | <0.001 | 0.47(0.40, 0.57) | <0.001 | 0.61(0.51, 0.74) | <0.001 |
| Quartile 4 | 0.42(0.35, 0.51) | <0.001 | 0.43(0.36, 0.52) | <0.001 | 0.59(0.48, 0.72) | <0.001 |
| P for trend |  | <0.001 |  | <0.001 |  | <0.001 |
| **30-day ICU mortality** |  |  |  |  |  |  |
| log2-ALI (continuous) | 0.81（0.77, 0.85） | <0.001 | 0.81（0.77, 0.86） | <0.001 | 0.90（0.85, 0.96） | <0.001 |
| log2-ALI (quartiles) |  |  |  |  |  |  |
| Quartile 1 | Ref |  | Ref |  | Ref |  |
| Quartile 2 | 0.72(0.59, 0.88) | 0.001 | 0.74(0.60, 0.89) | 0.002 | 0.84(0.69, 1.03) | 0.090 |
| Quartile 3 | 0.53(0.43, 0.66) | <0.001 | 0.54(0.44, 0.67) | <0.001 | 0.75(0.60, 0.93) | 0.010 |
| Quartile 4 | 0.46(0.36, 0.57) | <0.001 | 0.46(0.37, 0.58) | <0.001 | 0.66(0.53, 0.84) | <0.001 |
| P for trend |  | <0.001 |  | <0.001 |  | <0.001 |

Model 1: Unadjusted.

Model 2: Adjusted by age, gender, and race

Model 3: Adjusted by age, gender, race, heart rate, SBP, DBP, RR, SpO2, temperature, Hb, PLT, sodium, potassium, calcium, chloride, Total bilirubin, ALT, AST, BUN, Cr, aki, COPD, tumor, CKD, MV, SOFA score, APS-III score

## Supplementary Table S4

**Table S4** Sensitivity Analysis -3

| **Variables** | **Model 1** | | **Model 2** | | **Model 3** | |
| --- | --- | --- | --- | --- | --- | --- |
|  | **HR(95% CI)** | **P-value** | **HR(95% CI)** | **P-value** | **HR(95% CI)** | **P-value** |
| **30-day all-cause mortality** |  |  |  |  |  |  |
| log2-ALI (continuous) | 0.79（0.76, 0.82） | <0.001 | 0.80（0.76, 0.83） | <0.001 | 0.87（0.83, 0.91） | <0.001 |
| log2-ALI (quartiles) |  |  |  |  |  |  |
| Quartile 1 | Ref |  | Ref |  | Ref |  |
| Quartile 2 | 0.71(0.61, 0.82) | <0.001 | 0.72(0.62, 0.84) | <0.001 | 0.80(0.68, 0.93) | 0.004 |
| Quartile 3 | 0.47(0.40, 0.56) | <0.001 | 0.48(0.41, 0.57) | <0.001 | 0.63(0.53, 0.75) | <0.001 |
| Quartile 4 | 0.44(0.37, 0.52) | <0.001 | 0.45(0.38, 0.53) | <0.001 | 0.62(0.52, 0.74) | <0.001 |
| P for trend |  | <0.001 |  | <0.001 |  | <0.001 |
| **30-day ICU mortality** |  |  |  |  |  |  |
| log2-ALI (continuous) | 0.82（0.78, 0.86） | <0.001 | 0.82（0.78, 0.87） | <0.001 | 0.91（0.86, 0.96） | <0.001 |
| log2-ALI (quartiles) |  |  |  |  |  |  |
| Quartile 1 | Ref |  | Ref |  | Ref |  |
| Quartile 2 | 0.75(0.63, 0.90) | 0.002 | 0.76(0.64, 0.91) | 0.003 | 0.84(0.70, 1.01) | 0.062 |
| Quartile 3 | 0.53(0.43, 0.64) | <0.001 | 0.54(0.44, 0.66) | <0.001 | 0.73(0.59, 0.89) | 0.003 |
| Quartile 4 | 0.49(0.40, 0.60) | <0.001 | 0.50(0.41, 0.61) | <0.001 | 0.71(0.57, 0.88) | 0.002 |
| P for trend |  | <0.001 |  | <0.001 |  | <0.001 |

Model 1: Unadjusted.

Model 2: Adjusted by age, gender, and race

Model 3: Adjusted by age, gender, race, heart rate, SBP, DBP, RR, SpO2, temperature, Hb, PLT, sodium, potassium, calcium, chloride, Total bilirubin, ALT, AST, BUN, Cr, aki, COPD, tumor, CKD, MV, SOFA score, APS-III score, WBC

## Supplementary Table S5

**Table S5** Sensitivity Analysis -4

| **Variables** | **Model 1** | | **Model 2** | | **Model 3** | |
| --- | --- | --- | --- | --- | --- | --- |
|  | **OR(95% CI)** | **P-value** | **OR(95% CI)** | **P-value** | **OR(95% CI)** | **P-value** |
| **30-day all-cause mortality** |  |  |  |  |  |  |
| log2-ALI (continuous) | 0.75（0.72, 0.79） | <0.001 | 0.76（0.72, 0.80） | <0.001 | 0.81（0.77, 0.86） | <0.001 |
| log2-ALI (quartiles) |  |  |  |  |  |  |
| Quartile 1 | Ref |  | Ref |  | Ref |  |
| Quartile 2 | 0.65(0.54, 0.78) | <0.001 | 0.67(0.55, 0.80) | <0.001 | 0.71(0.58, 0.87) | <0.001 |
| Quartile 3 | 0.41(0.33, 0.49) | <0.001 | 0.42(0.34, 0.51) | <0.001 | 0.52(0.42, 0.64) | <0.001 |
| Quartile 4 | 0.37(0.30, 0.45) | <0.001 | 0.38(0.31, 0.47) | <0.001 | 0.49(0.39, 0.62) | <0.001 |
| P for trend |  | <0.001 |  | <0.001 |  | <0.001 |
| **30-day ICU mortality** |  |  |  |  |  |  |
| log2-ALI (continuous) | 0.80（0.76, 0.85） | <0.001 | 0.80（0.76, 0.85） | <0.001 | 0.87（0.81, 0.92） | <0.001 |
| log2-ALI (quartiles) |  |  |  |  |  |  |
| Quartile 1 | Ref |  | Ref |  | Ref |  |
| Quartile 2 | 0.72(0.59, 0.89) | 0.002 | 0.73(0.60, 0.90) | 0.003 | 0.78(0.62, 0.98) | 0.035 |
| Quartile 3 | 0.49(0.39, 0.61) | <0.001 | 0.50(0.40, 0.62) | <0.001 | 0.64(0.50, 0.82) | <0.001 |
| Quartile 4 | 0.45(0.36, 0.57) | <0.001 | 0.46(0.37, 0.58) | <0.001 | 0.61(0.47, 0.79) | <0.001 |
| P for trend |  | <0.001 |  | <0.001 |  | <0.001 |

OR Odds ratio

Model 1: Unadjusted.

Model 2: Adjusted by age, gender, and race

Model 3: Adjusted by age, gender, race, heart rate, SBP, DBP, RR, SpO2, temperature, Hb, PLT, sodium, potassium, calcium, chloride, Total bilirubin, ALT, AST, BUN, Cr, aki, COPD, tumor, CKD, MV, SOFA score, APS-III score
